# Management of Lower Limb Ischemia During Operative Repair of Acute Type A Aortic Dissection by Distal Crossover Grafts: a Case Series

**DOI:** 10.21470/1678-9741-2019-0259

**Published:** 2020

**Authors:** Thomas Theologou, Amer Harky, Matthew Shaw, Hazim Eltyeb, Walid Elbakbak, Mostafa Snosi, Deborah Harrington, Manoj Kuduvalli, Aung Oo, Mark Field

**Affiliations:** 1 Department of Cardiothoracic Surgery, Liverpool Heart and Chest Hospital, Liverpool, United Kingdom.

**Keywords:** Aneurysm, Dissection, Ischemia, Peripheral Vascular Diseases, Extremities, Amputation, Early Diagnosis, Referral and Consultation, Surgeons

## Abstract

**Objective:**

To describe our experience of nine patients with extra-anatomical bypass for clinically ischemic distal limb during repair of acute Type A aortic dissection (ATAAD).

**Methods:**

We retrospectively examined a series of nine patients who underwent surgery for ATAAD. We identified a subset of the patients who presented with concomitant radiographic and clinical signs of lower limb ischemia. All but one patient (axillobifemoral bypass) underwent femorofemoral crossover grafting by the cardiac surgeon during cooling.

**Results:**

One hundred eighty-one cases of ATAAD underwent surgery during the study period with a mortality of 19.3%. Nine patients had persistent clinical evidence of lower limb ischemia (4.9%) and underwent extra-anatomical bypass during cooling. Two patients underwent additional fasciotomies. Mean delay from symptoms to surgery in these nine patients was 9.5 hours. Two patients had bilateral amputations despite revascularisation and, of note, had long delays in presentation for surgery (> 12 hours). There were no mortalities during these inpatient episodes. Outpatient radiographic follow-up at the first opportunity demonstrated 100% patency.

**Conclusion:**

Our experience suggests that, during complicated aortic dissection, limb ischemia may have a devastating outcome including amputation when diagnosis and referral are delayed. Early diagnosis and surgery are crucial in preventing this potentially devastating complication.

**Table:** 

Abbreviations, acronyms & symbols	
AKA	= Above-knee amputation
ATAAD	= Acute Type A aortic dissection
CABG	= Coronary artery bypass grafting
CABGX2	= Two coronary artery bypass graftings
CTA	= Computed tomography angiogram
CVA	= Cerebrovascular accident
CVVH	= Continuous venovenous hemofiltration
F	= Female
FET	= Frozen elephant trunk
ITU	= Intensive therapy unit
M	= Male
NYHA	= New York Heart Association
TOE	= Transoesophageal echocardiography
UK	= United Kingdom

## INTRODUCTION

Recognising and treating successfully an emergency complicated acute Type A aortic dissection (ATAAD) is a real challenge despite the recent advances in aortic surgery. ATAAD is a lethal condition with a mortality rate of as high as 50-60% in the first 48 hours of presentation if left untreated. A complicated ATAAD with malperfusion syndrome carries significant mortality and morbidity even before admission for definitive management^[1,2]^.

The urgent repair of ATAAD addresses the correction of the proximal extent of the pathology. However, there are rare conditions in which distal dissection can result in malperfusion of vital organs, the abdomen, or the lower limbs, and the prognosis in such cohort is poor. Clinically evident lower limb ischemia is a rare event and it is sometimes difficult to recognise as the diagnosis is obscured by general hypoperfusion during the acute dissection and the general status of the patient. Despite its rarity, it can still affect 5-15% of the patients, and it is caused by malperfusion from the dissection flap occluding or inducing thrombosis in the distal aortic branch arteries of the lower limbs^[3,4]^.

Traditionally, all patients with ATAAD are typically treated with an urgent sternotomy and graft replacement of the ascending aorta^[5]^. Persistent malperfusion after proximal aortic repair can be treated with open or endovascular revascularization techniques. Nowadays, distal ischemia is the first indication for frozen elephant trunk (FET) technique or procedures related to the Petticoat concept in order to reduce the false lumen effect, expand the true lumen, and respect the patency of the aortic branches without compromising the spinal cord perfusion^[6]^. Open techniques, if there is distal limb ischemia, are the conventional extra-anatomical bypasses, like femorofemoral crossover bypass, or, if both limbs are in danger, an axillobifemoral bypass^[7]^. However, due to advances in endovascular techniques, a percutaneous aortic flap fenestration or branch artery stenting can be performed in order to improve the peripheral perfusion prior to definitive proximal aortic repair^[8]^. The rationale behind using the new endovascular techniques is to improve the blood flow to the end-organ, which is at risk of ischemic injury, and then to carry on with the open repair of the dissecting aorta. This is because of the reported higher postoperative mortality of patients with end-organ or limb ischemia^[9]^.

This work describes our experience on treating complicated ATAAD with extra-anatomical bypass for clinically ischemic distal limb in our aortic centre in the United Kingdom (UK).

## METHODS

All patients who presented to our centre for emergency repair with ATAAD have been retrospectively examined. As an aortic specialist centre, we accepted acute aortic pathologies referred from areas of the North West of England, North Wales, and a small number of places across the UK that were directed to our centre. We identified a subset of patients who presented with concomitant radiographic and clinical signs of lower limb ischemia at induction.

Patients included in the study had an ATAAD with a subintimal tear involving the ascending aorta or the arch and it was considered to be acute if symptoms occurred within 14 days of admission. Patients with acute intramural hematoma in the ascending aorta were included, whereas those with chronic dissection were excluded. Diagnosis was established using an electrocardiogram-gated contrast-enhanced computed tomography scanning and pre- or intraoperative echocardiography to assess for intimal tear on the proximal aorta and evidence of aortic regurgitation or acute pericardial collection with or without evidence of an imminent tamponade. All included patients had their diagnosis confirmed at the operation during a transoesophageal echocardiography (TOE) assessment and evaluation, if time permitted. Limb ischemia was diagnosed by clinical signs and symptoms and was confirmed on the computed tomography angiogram (CTA) of the aorta where there was little or no contrast seen in distal aortic branches or if a dissection and/or thrombus occluded the distal aortic iliac or femoral vessels. Most of the patients had a CTA performed in the referring hospitals, but there were several patients who were transferred from outside hospitals that had only a few imaging tests to evaluate the iliac or femoral arteries. In some of these patients, computed tomography images were typically brought with the patient on a computer disk and sometimes included only thoracic aortic views. Due to the urgency of the operation and the risk of contrast-induced nephropathy, CTAs were not repeated on these patients but we did assess the pulses with a Doppler scan to see if they were present or not, prior to deciding on the operative and cannulation strategy. Primary outcome measures included in-hospital mortality, need for peripheral revascularization, limb loss, cerebrovascular accident (CVA), and renal failure. Intensive therapy unit (ITU) stay, hospital stay, and other morbidities have been analysed and recorded in the study.

### Operative Technique

Our treatment pathway consisted of an emergency operation with cardiopulmonary bypass, deep hypothermic circulatory arrest, antegrade cerebral perfusion, antegrade and retrograde myocardial protection^[10,11]^. Cardiopulmonary bypass was initiated via direct open axillary cannulation if there was a femoral artery pulse deficit, ascending or arch direct aortic true lumen cannulation using the epiaortic scanning, and in some extreme cases cannulation of the left ventricular apex with TOE guidance to pass the aortic valve if no other access was available.. Depending on the case and the anatomical involvement of the aortic dissection, the aortic cannulation sites varied, and they are presented on Table 1. Although the cooling period is usually less than 40 minutes, adequate femoral arterial inflow is monitored by cerebral oximetry as well as bladder and nasopharyngeal temperatures. Venous cannulation was in the right atrium with a two-stage cannulation or femoral vein. Superior and inferior vena cava control was obtained, and the patient was cooled. Continuous retrograde cold blood cardioplegia solution was administered via coronary sinus, whereas intermittent antegrade administration of cardioplegia was delivered via coronary ostia after opening the aorta. Venting was achieved via the right superior pulmonary vein, and venting and de-airing at the end of the operation were achieved through the ascending aorta. After isoelectric electroencephalography, usually at 18-20 °C, bypass was discontinued, and circulation was arrested. During the cooling time, the femorofemoral crossover bypass had been constructed in order to achieve perfusion to the lower limb, given that it was in danger. The ascending aorta was then opened without cross-clamping and antegrade cerebral perfusion was started via the neck vessels. After assessing the arch and all the neck vessels, the ascending aorta was replaced with a Dacron graft and the intimal tear was resected, regardless of whether it was an entry or re-entry tear. In these series of patients, most tears were in the ascending aorta, and few were on the aortic arch. Therefore, a hemiarch replacement was sufficient to repair the dissection. If the intimal defect was within the transverse aortic arch, most of the entire arch was replaced. After completion of the distal anastomosis, retrograde cerebral perfusion was stopped and antegrade flow was initiated. With the patient in the Trendelenburg position, air and debris were flushed from the aorta through the open graft. Antegrade flow was initiated via a side-arm cannula through the distal anastomosis with the proximal graft clamped. Resuspension of the aortic valve or aortic valve replacement or aortic root replacement was performed in the patients in the study. In some cases, an aortic valve replacement and a coronary artery bypass surgery were undertaken concomitantly (see Table 2). The patient was warmed to 36 °C and the proximal anastomosis was completed. The patient was weaned from bypass, cannulas were removed, and anticoagulation was reversed. The lower limbs were again reassessed, and a Doppler scan was performed to assess the presence of the distal pulses. If there was a doubt about the pulses or the limb was ischemic, then exposure of the femorofemoral bypass was done and an embolectomy or remodelling of the anastomosis was performed before the patient transferred to the intensive care. In one of the cases, despite correcting the dissection, the legs were still ischemic, and an axillobifemoral bypass was performed to salvage both limbs.

**Table 1:** Characteristics of the nine patients operated with femorofemoral bypass.

		Important notes	Cannulation	Leg site	Complications	Notes
1	M	Bovine arch	Left ventricular cannulation	Ischemic left leg	-	Tamponade
2	M	Normal arch	Right axillary cannulation	Ischemic right leg	-	
3	F	Bovine arch	Right femoral cannulation	Ischemic left leg	Fasciotomy	
4	M	Normal arch	Right axillary cannulation	Ischemic right leg	Paraparesis, reoperation for bleeding from the femoral bypass	
5	M	? Marfan	Right axillary cannulation	Ischemic right leg	Right kidney was already ischemic on presentation	
6	M	Wegener's granulomatosis	Direct true lumen cannulation	Ischemic right leg		
7	F	Delay of presentation	Innominate artery cannulation	Both legs were ischemic	Left leg amputation	Axillary artery dissected
8	F	Delay of presentation	Right axillary & femoral cannulation	Both legs were ischemic	Had bowel ischemia, laparotomy & colostomy, bilateral AKA, fasciotomies	Presented with paraesthesia in both legs, paraplegia, and metabolic acidosis
9	F	Normal arch	Right axillary cannulation	Ischemic left leg	Had CABGX2	Persistent leg ischemia despite femorofemoral bypass, so axillobifemoral bypass was performed

AKA=above-knee amputation; CABGX2=two coronary artery bypass graftings; F=female; M=male

**Table 2:** Patients' demographics, co-morbidities, and operative details.

Variables	All acute Type A dissections	All femoro-femoral bypasses
	(n=181)	(n=9)
Age at operation (years)	61 (52-70)	44 (41-55)
Female gender	61 (33.7%)	4 (44.4%)
Comorbidities		
Body mass index (kg/m^2^)	27.2 (24.2-30.1)	27.8 (24.4-29.5)
Left ventricular ejection fraction < 50%	28 (15.5%)	1 (11.1%)
NYHA class ≥ III	26 (14.4%)	0 (0)
Current smoker	43 (23.8%)	4 (44.4%)
Diabetes	6 (3.3%)	1 (11.1%)
Hypercholesterolemia	39 (21.5%)	1 (11.1%)
Hypertension	113 (62.4%)	1 (11.1%)
Cerebrovascular disease	12 (6.6%)	0 (0)
Respiratory disease	30 (16.6%)	0 (0)
Peripheral vascular disease	13 (7.2%)	1 (11.1%)
Renal dysfunction	36 (19.9%)	3 (33.3%)
Previous cardiac surgery	8 (4.4%)	1 (11.1%)
Priority		
Emergency procedure	154 (85.1%)	8 (88.9%)
Salvage procedure	7 (3.9%)	1 (11.1%)
Extent of aorta replaced		
Aortic root	91 (50.3%)	4 (44.4%)
Ascending aorta	171 (94.5%)	8 (88.9%)
Aortic arch	76 (42.0%)	6 (66.7%)
Concomitant procedures		
Aortic valve replacement	105 (58.0%)	5 (55.6%)
CABG	28 (15.5%)	1 (11.1%)
Operative times		
Circulatory arrest	45 (34-58)	33.5 (20.8-49)
Cardiopulmonary bypass	333 (271-392)	351 (333.5-415.5)
Aortic cross-clamp	180 (131-231)	196 (139-218)

CABG=coronary artery bypass grafting; NYHA=New York Heart Association

## RESULTS

During this 8-year period, 181 cases underwent emergency surgery for correction of ATAAD (Table 2). The patients’ mean age was 61 years (range 52-70) with predominance of male (66.3%) and hypertensive (62.4%) patients. Several of them already suffered from renal failure (19.9%) and were current smokers (23.8%), with 7.2% diagnosed with symptomatic peripheral vascular disease. Emergency procedures were performed in 85.1% of the cases and salvage was in 3.9% of patients, with the rest operated as semi-urgent procedures due to the stability of their condition. Most of the patients had an ascending aortic replacement (94.5%), 50% had aortic root replacement and 42% had aortic arch replacement. About 58% of the patients had an aortic valve replacement and only 15.5% had a concomitant coronary artery bypass grafting. Mean time of circulatory arrest was 45 minutes (range 34-58). Regarding the outcomes of the whole group of ATAAD repaired, the mean ITU stay was six days (range 3-13), with a postoperative stay of 14 days (range 9-26). Acute renal failure requiring continuous venovenous hemofiltration (CVVH) dialysis occurred in 20.4% of the patients, CVA in 11% of the patients, with paraplegia in 0.6%. In-hospital mortality was 19.3% (Table 3).

**Table 3:** Patients' outcome.

Variables	All acute Type A dissections	All femoro-femoral bypasses
	(n=181)	(n=9)
ITU stay (days)	6 (3-13)	10 (3-32)
Postoperative stay (days)	14 (9-26)	21 (14.5-49.5)
Acute renal failure	37 (20.4%)	3 (33.3%)
Deep sternal wound infection	1 (0.6%)	0 (0)
Reoperation	40 (22.1%)	4 (44.4%)
Reoperation for bleeding	22 (12.2%)	1 (11.1%)
CVA	20 (11.0%)	1 (11.1%)
Paraplegia	1 (0.6%)	1 (11.1%)
Reoperation for bleeding from the femorofemoral anastomosis	-	1 (11.1%)
Reoperation for tamponade	9 (4.97%)	1 (11.1%)
In-hospital mortality	35 (19.3%)	0 (0)

CVA=cerebrovascular accident; ITU=intensive therapy unit

The demographics of the subgroup of nine patients who presented with acute limb ischemia is presented on Table 2. It is recorded that 44.4% of the patients are of female gender and underwent extra-anatomical bypass during cooling. Of these patients, 44.4% were active smokers and 33.3% already had renal dysfunction. Emergency procedure was performed in 88.9% of the patients, with only one having a salvage procedure, who was admitted directly to theatre with a cardiac tamponade and ischemic leg. As in the whole group recorded earlier as well in this group of patients, 88.9% had ascending aortic replacement, 66.7% had aortic arch replacement, and 44.4% had aortic root replacement; 55.6% of patients had an aortic valve replacement and only one had a concomitant coronary artery bypass grafting. The ischemic time was 33.5 minutes (range 20.8-49). Bypass and cross-clamp time didn’t differ from the whole group of the 181 patients. Median delay from symptoms to surgery in these nine patients was 9.5 hours.

The mean ITU stay was 10 days (range 3-32) for the patients treated with femorofemoral bypass and the mean postoperative stay was 21 days (range 14.5-49.5). Renal failure requiring CVVH occurred in 33.3% of the patients in this group, with resolution and normalization of their creatinine on the day of discharge. There was only one patient with CVA and one patient with paraplegia. Two patients underwent additional fasciotomies for treatment of compartment syndrome. These two patients who had fasciotomies had lower limb amputations despite successful revascularisation. Of note, both patients had long delays from their diagnosis to the admission for surgery (> 12 hours). Two patients had concomitant malperfusion to the bowel, requiring a laparotomy, and one patient had already developed an ischemic right kidney. There was one patient who already suffered from Wegener’s granulomatosis and one patient who had Marfanoid appearances clinically. It is interesting that two of the nine patients who had a bovine arch had left leg ischemia. There were no in-hospital deaths. Outpatient radiographic follow-up at the first opportunity demonstrated 100% patency on a CTA and after 3, 6, and 12 months.

## DISCUSSION

The association between ATAAD and malperfusion of distal organs or limbs is not a common phenomenon, however when it is present, it carries significant morbidity and mortality^[10,11]^. Depending on which organ is malperfused, the time to recognise and act on it is crucial for the patient’s outcome. Malperfused intra-abdominal organs, heart, or brain compromise the outcome of the patient imminently with high mortality, as it has been recorded in the International Registry of Acute Aortic Dissection - IRAD study^[12]^. However, when isolated limbs in the periphery, as the aortic branches of the iliofemoral axis, are malperfused, there is a limited, but more, time to recognise it in order to save the patient’s life and limbs. In our study, whilst a small, non-randomised cohort series of nine patients, our treatment approach showed no inpatient mortality, but it is consistent with devastating outcomes (amputations) when combined with delayed diagnosis and referral for definitive treatment.

Optimal management for ATAAD is the proximal entry closure, and when this is established, theoretically, the distal false lumen can be thrombosed with no progression of the dissection to the distal branches of the aorta. However, the remaining type B dissection can progress with increasing of the false lumen. On the other hand, when the tear is in the arch and if there is evidence of distal malperfusion, the FET method is indicated ^[13]^.In order to treat these complex lesions of the thoracic aorta, the FET technique has become increasingly common as studies have shown optimal results^[14]^. In a recent review by Ma et al.^[15]^, the early mortality rate ranged from 6.4% to 15.8%. The recent data from the E-Vita registry showed that the early results were comparable between aortic dissection and chronic degenerative aneurysm, with in-hospital mortality rates of 17.1% and 13.2%, respectively^[16]^. It’s already an evidence that a hybrid approach involving full arch replacement and insertion of an endovascular stent graft into the descending aorta in the setting of ATAAD has been advocated in patients with distal malperfusion^[17,18]^.

Recognition of the ATAAD is based on clinical and radiological findings. But recognition of additional malperfusion syndrome is more challenging, requiring careful attention to the clinical signs and symptoms from the abdomen or limbs. Pain and acidosis with high lactates suggest that the organ is in danger, but it is not very specific, due to the whole hypoperfusion syndrome and stress of the organism during acute presentation of the dissection^[19]^. In the study by Suliman et al.^[20]^, they stated that lactates are an index of aggravation and suspicion. However, as it is not specific, more tests are required to complete the correct diagnosis.

In the cases of central aortic repair, limb ischemia can be managed through revascularisation via a femorofemoral bypass or an axillofemoral bypass during the procedure. Endovascular stenting without entry closure for ATAAD has the risk of cardiac tamponade. However, in specialist centres, as it has been recorded in the literature, the endovascular approach has been successful to treat imminent organ or limb malperfusion prior correcting the proximal aortic tear^[21,22]^. In this study, guided by CTA and the patients’ symptomatology after high suspicion of malperfusion to the lower limbs and bedside ultrasound Doppler to assess the lower limb pulses, we treated these nine patients with femorofemoral bypass, if there was one lower limb malperfusion, and axillobifemoral bypass, if both lower limbs were in danger.

Estrera et al.^[23]^ advocated the same technique in their recent series. We choose to do this during cooling time as there was plenty of time to correct the defect and re-establish antegrade flow to the lower limb as its more physiological after reperfusion via the side arm in order to save the limb. As we can see in this series of patients, we didn’t have mortality, however significant morbidities, including amputations and renal failure, have been recorded. This is due to the delay of presentation (> 12 hours, in some cases) and pre-existing renal impairment of some other patients. In the whole group of 181 patients, mortality was high, but this occurs because, as it has been already reported in the literature by Estrera et al.^[23]^, malperfusion syndrome, specifically concomitant bowel ischemia and multiple organ failure and CVAs, was high in this cohort of patients. For the group of nine patients in whom the analysis has been performed, only one patient presented with intestinal malperfusion requiring bowel resection and laparotomy. Expeditious and immediate repair of the distal ischemia saved the lower limbs despite the malperfusion syndrome presented.

## CONCLUSION

We can conclude in this study that recognising distal limb ischemia at the time of an ATAAD is important as it can portend to poor outcomes. Our experience suggests that this form of malperfusion may have devastating outcomes, including amputation, when diagnosis and referral are delayed. While a small, non-randomised experience, our approach demonstrated no hospital mortality in this high-risk group of patients. Early diagnosis and surgery are crucial in preventing this potentially devastating complication.

**Table:** 

Authors' roles & responsibilities	
TT	Substantial contributions to the conception, design of the work; the acquisition, analysis, interpretation of data for the work; drafting the work and revising it critically for important intellectual content; final approval of the version to be published
AH	Substantial contributions to the acquisition, analysis, or interpretation of data for the work; drafting the work or revising it critically for important intellectual content; final approval of the version to be published
MS	Substantial contributions to the design of the work; or the acquisition, analysis, or interpretation of data for the work; drafting the work or revising it critically for important intellectual content; final approval of the version to be published
HE	Substantial contributions to the design of the work; or the acquisition, analysis, or interpretation of data for the work; drafting the work or revising it critically for important intellectual content; final approval of the version to be published
WE	Substantial contributions to the acquisition, analysis, or interpretation of data for the work; drafting the work or revising it critically for important intellectual content; final approval of the version to be published
MS	Substantial contributions to the acquisition, analysis, or interpretation of data for the work; drafting the work or revising it critically for important intellectual content; final approval of the version to be published
DH	Substantial contributions to the conception or design of the work; drafting the work or revising it critically for important intellectual content; final approval of the version to be published
MK	Substantial contributions to the conception or design of the work; drafting the work or revising it critically for important intellectual content; final approval of the version to be published
AO	Substantial contributions to the conception or design of the work; or the acquisition, analysis, or interpretation of data for the work; drafting the work or revising it critically for important intellectual content; final approval of the version to be published
MF	Substantial contributions to the conception or design of the work; or the acquisition, analysis, or interpretation of data for the work; drafting the work or revising it critically for important intellectual content; final approval of the version to be published
